# Growth-Associated Succession and Persistence of Gill and Skin Mucus Microbiota in Cultured *Larimichthys crocea*

**DOI:** 10.3390/biology15211922

**Published:** 2026-10-23

**Authors:** Fang Liu, Ding Li, Peixin Wu, Shuhan Lin, Jie Yu, Fangyu Yuan, Weiqing Huang, Kunhuang Han

**Affiliations:** 1 College of Marine Sciences, Ningde Normal University, Ningde 352100, China; lf_ndsy@163.com (F.L.); wupeixinxz@163.com (P.W.); llllinyy@163.com (S.L.); yu20250626@163.com (J.Y.); 2 Fujian Provincial University Engineering Research Center for Deep Processing of Mindong Aquatic Products, Ningde 352100, China; 3 Haixi Public Platform for Marine Characteristic Germplasm Resources and Biological Products Development, Ningde 352100, China; 4 School of Medicine, Fuzhou University, Fuzhou 350108, China; t25116@fzu.edu.cn

**Keywords:** *Larimichthys crocea*, gill-associated microbiota, skin mucus, microbial succession, persistent ASVs, aquaculture

## Abstract

External mucosal surfaces form major interfaces between fish and the aquatic environment, yet their microbial succession during host growth remains incompletely resolved. We characterized the gill and skin mucus microbiota of cultured *Larimichthys crocea* at three successive body-size stages (5–8 g, 39–46 g, and 160–162 g) using 16S rRNA gene amplicon sequencing. At each stage, 15 fish were sampled, and material from three fish was pooled to generate one pooled sample, yielding five pooled samples for each host-associated group. Four samples of truck-delivered seawater supplied by the same provider used throughout cultivation were included as an environmental reference. Rarefaction curves approached saturation, and amplicon sequence variant (ASV)-level community structure differed significantly among the seven groups (ANOSIM, R = 0.7521, *p* = 0.001). Early-stage gill and skin mucus communities occupied clearly separated regions of ordination space and showed distinct dominant taxa, whereas large-stage gill and skin mucus communities showed reduced separation and greater similarity in dominant taxonomic composition. At the genus level, 2013Ark19i characterized early-stage gills, *Vibrio* and *Photobacterium* were prominent in early-stage skin mucus, and *Methylobacterium* increased markedly in both large-stage mucosal communities. Although 17 phyla, 67 families, and 69 genera were shared across all groups, only 30 ASVs were universally shared, indicating extensive fine-scale turnover. Dataset-wide prevalence classification further showed that persistent ASVs contributed an increasing fraction of community abundance during growth, rising from 21.93% to 75.48% in gills and from 12.77% to 67.61% in skin mucus. Middle-stage skin mucus contained 1387 group-specific ASVs and the highest transient-ASV contribution among host-associated groups, indicating particularly extensive turnover. Overall, external mucosal microbiota exhibited tissue-specific and non-linear growth-associated reorganization, with pronounced intermediate-stage turnover and increasing abundance dominance of persistent ASVs during later growth.

## Simple Summary

Fish gills and skin mucus are continuously exposed to the aquatic environment but provide different biological surfaces for microbial colonization. We examined the gill and skin mucus microbiota of cultured *L. crocea* at three successive body-size stages and included supplied seawater from the same provider as an environmental reference. The two mucosal habitats were clearly different at the smallest body-size stage, while their communities showed reduced separation at the largest stage. Middle-stage skin mucus contained particularly many stage-specific and transient microbial members, indicating extensive community turnover. In contrast, later-stage gill and skin mucus communities contained much larger abundance contributions from microbial populations that were repeatedly detected across the full dataset. These findings show that external mucosal microbiota undergo substantial growth-associated reorganization and that gill and skin mucus do not follow identical trajectories during fish growth.

## 1. Introduction

Fish-associated microbiota are integral components of the host–environment interface and are increasingly recognized as important contributors to fish physiology, nutrient utilization, immune homeostasis, and environmental adaptation [1,2,3,4]. Unlike free-living microbial communities in surrounding water, host-associated microbiota are shaped by both environmental exposure and local host conditions, including tissue characteristics, mucus composition, nutrient availability, immune regulation, and physicochemical properties [1,2,5]. Consequently, fish mucosal microbiota frequently form assemblages that differ from environmental microbial communities despite continuous exposure to the aquatic microbial pool [6,7,8].

The gill and skin mucus represent two major external mucosal habitats. Both are in direct contact with water, but they differ substantially in physiological function and local habitat characteristics [5,9,10,11]. The gill is a highly specialized organ responsible for gas exchange, osmoregulation, ion transport, acid–base regulation, and nitrogenous waste excretion [12], whereas skin mucus constitutes a continuously renewed external barrier at the body surface [9,10]. These contrasting microhabitats can support different microbial communities, and previous studies have reported differentiation among gill, skin, gut, and environmental microbiota within the same fish species or habitat [5,9,10,11,13].

Fish-associated microbial communities can also change during host growth. Age and growth stage covary with body size, feeding intensity, metabolism, immune development, mucus secretion, epithelial properties, and cumulative environmental exposure, all of which may contribute to microbial succession [14,15,16,17,18]. Age-related microbial shifts have been reported in farmed Chinook salmon, Atlantic salmon, tilapia, and other teleosts [15,16,17,18]. In *L. crocea*, intestinal microbiota vary substantially among age classes [14,15]. However, developmental microbiome studies have focused mainly on the intestine, and considerably less is known about how external mucosal communities reorganize across later growth stages.

Skin mucus is a physiologically dynamic matrix rather than a static microbial substrate. Its soluble proteins, metabolites, and other biochemical components can vary with culture conditions, physiological stress, and physical disturbance, thereby altering the habitat available to surface-associated microorganisms [19]. Recent studies have demonstrated culture-system-associated variation in skin-mucus biomarkers and changes in both epidermal mucus composition and associated microbial communities following physical disturbance [20]. These observations provide important physiological context for interpreting temporal changes in skin-mucus microbiota and indicate that mucosal community variation may reflect both host-associated and environmental processes. External mucosal surfaces also constitute first-line interfaces between fish and their environment [21]. Understanding how their microbial communities reorganize across growth stages may provide ecological insight into mucosal barrier function, colonization resistance, and the potential exclusion of opportunistic microorganisms. However, because the present study is based on 16S rRNA gene profiles, these potential protective roles are considered ecological hypotheses rather than directly demonstrated microbial functions.

Microbial succession may involve not only taxonomic turnover but also changes in how consistently individual amplicon sequence variants (ASVs) occur across samples. Some ASVs are detected sporadically, whereas others recur across a large proportion of samples and can contribute disproportionately to community abundance. Classification of transient, intermittent, and persistent ASVs therefore provides an ecological dimension complementary to conventional diversity and taxonomic analyses. For external mucosal habitats that remain directly exposed to water, changes in the abundance contribution of recurrent ASVs may be particularly informative for understanding growth-associated community organization [7,8,22].

*L. crocea* is an economically important marine aquaculture species in China [23,24]. Undergoes substantial changes in body size, feeding demand, physiology, and external mucosal characteristics during cultivation. Its gill and skin surfaces are continuously exposed to the aquatic environment and represent important interfaces for respiration, osmoregulation, barrier protection, and host–microbe interactions. Recent studies have demonstrated clear differentiation among skin, gill, and gut microbiota in this species [13], while our previous work showed that *L. crocea* mucosal microbiota differ from environmental water communities and respond differently to contrasting aquaculture conditions [25]. In the present study, we examined gill and skin mucus microbiota at three body-size stages (5–8 g, 39–46 g, and 160–162 g). Four samples of the seawater supplied to the culture facility by the same provider used throughout cultivation were included as an environmental reference rather than as stage-matched tank-water samples. We evaluated growth-associated changes in community structure, taxonomic composition, shared and stage-specific membership, and dataset-wide microbial persistence to determine how external mucosal microbiota reorganize across the sampled growth sequence.

## 2. Materials and Methods

### 2.1. Experimental Design and Sample Collection

All fish originated from the same cultured population and were maintained in the same culture pond throughout the cultivation period. The same formulated diet was used throughout cultivation, and feed composition remained unchanged among the three sampling stages. Only feeding quantity was adjusted according to fish growth and biomass. Detailed contemporaneous records of stocking density and physicochemical water parameters were not retained for all three sampling periods. Consequently, possible temporal variation in rearing conditions could not be quantitatively separated from growth-stage-associated changes. The three sampling groups were therefore defined by successive body-size classes rather than by precisely recorded chronological age. Exact chronological ages and precise intervals between sampling events were not retained in the available records.

Gill samples from the early, middle, and large stages were designated DHYEGS, DHYMGS, and DHYLGS, respectively, whereas the corresponding skin mucus samples were designated DHYEGTB, DHYMGTB, and DHYLGTB. At each growth stage, 15 apparently healthy fish were randomly selected and divided into five non-overlapping subsets of three fish. For each tissue, material from the same three fish within each subset was pooled to generate one composite sample, yielding five paired pooled gill and skin-mucus samples per growth stage. Thus, gill and skin-mucus samples with the same replicate number originated from the same subset of three fish. No individual fish contributed to more than one pool. The pooled sample, rather than the individual fish, was treated as the analytical unit. Pooling was used to characterize group-level microbial profiles but necessarily reduces information on inter-individual microbiome variability.

Fish were euthanized using tricaine methanesulfonate (MS-222; 200 mg L^−1^) before tissue collection. Skin mucus was collected aseptically from the abdominal body surface, after which gill tissue was excised using sterile scissors and forceps. Skin mucus was collected aseptically from a standardized abdominal body-surface region using sterile cotton swabs while avoiding contact with the anus and gill region. Sampling instruments were sterilized or replaced between pooled samples to minimize cross-contamination. Gill tissues were subsequently excised using sterile scissors and forceps. In the present study, “gill-associated microbiota” refers to microorganisms recovered from excised gill tissue, including microorganisms associated with the gill surface and tissue, rather than a separately isolated gill-mucus fraction. Sampling instruments were sterilized or replaced between pooled samples to minimize cross-contamination. All host-associated samples were transferred immediately into sterile cryogenic tubes, rapidly frozen in liquid nitrogen, transported under cold-chain conditions, and stored at −80 °C until DNA extraction.

### 2.2. Supplied Seawater Sampling and Genomic DNA Extraction

Seawater used in the culture system was delivered to the facility by tanker truck from the same provider throughout cultivation. Four supplied-seawater samples, designated DHY_ST1–DHY_ST4, were collected from four independent tanker-delivery events and were included as environmental reference samples. These samples were not collected synchronously with the three fish-sampling events and therefore were not treated as stage-matched culture-water samples. Consequently, comparisons involving DHYST were used to provide environmental context rather than to infer contemporaneous or stage-specific host–water microbial exchange.

Approximately 2 L of each seawater sample was filtered through a sterile 0.22 μm membrane to concentrate microbial biomass. Microbial genomic DNA was extracted using the DNeasy PowerSoil Pro Kit (QIAGEN GmbH, Hilden, Germany) according to the manufacturer’s protocol. Gill tissues were aseptically homogenized before DNA extraction. DNA from skin mucus was extracted from the material collected on sterile cotton swabs, while DNA from seawater samples was extracted from microbial biomass retained on the filtration membranes.

The concentration of extracted DNA was determined using a Qubit 3.0 Fluorometer (Life Technologies, Thermo Fisher Scientific, Waltham, MA, USA) and the Equalbit 1× dsDNA HS Assay Kit (Vazyme, Nanjing, China). DNA integrity and purity were further assessed using 1.0% agarose gel electrophoresis and a NanoDrop ND-2000 spectrophotometer (Thermo Scientific, Waltham, MA, USA).

Negative controls were included throughout the laboratory workflow to monitor potential procedural contamination. Procedural blanks were included during seawater filtration and DNA extraction, and no-template negative controls were included during PCR amplification. These controls were processed alongside the experimental samples under the same laboratory conditions.

### 2.3. 16S rRNA Gene Amplification and Illumina Sequencing

The V4 region of the prokaryotic 16S rRNA gene was amplified using the primer pair 515FmodF (5′-GTGYCAGCMGCCGCGGTAA-3′) and 806R (5′-GGACTACHVGGGTWTCTAAT-3′). The V4 region was selected because the 515FmodF/806R primer pair provides broad coverage of prokaryotic 16S rRNA gene diversity and generates an amplicon length well suited to paired-end Illumina sequencing. The widespread use of this region in aquatic microbiome studies also facilitates comparison with previously reported microbial community datasets. PCR reactions were performed in a total volume of 20 μL containing 10 ng of template DNA, 0.8 μL of each primer (10 μM), 4 μL of 5× FastPfu buffer, 0.4 μL of FastPfu polymerase, 0.2 μL of bovine serum albumin, and 250 μM dNTPs. PCR cycling consisted of an initial denaturation at 95 °C for 3 min, followed by 29 cycles of denaturation at 95 °C for 30 s, annealing at 55 °C for 30 s, and extension at 72 °C for 45 s, with a final extension at 72 °C for 10 min. PCR products were examined by agarose gel electrophoresis, purified, quantified, and pooled in equimolar proportions before sequencing. Purified amplicons were sequenced using an Illumina MiSeq PE300 platform according to the standard protocols of Majorbio Bio-Pharm Technology Co., Ltd. (Shanghai, China).

### 2.4. Sequence Processing and Taxonomic Assignment

Raw sequencing reads were processed using the QIIME 2 (version 2020.2) pipeline [26]. Primer sequences were removed using Cutadapt (version 2.8) [27], followed by quality filtering, denoising, paired-end merging, and chimera removal using DADA2 (version 2020.2.0) [28]. Non-singleton amplicon sequence variants (ASVs) were retained. Representative ASV sequences were aligned using MAFFT (version 7.310) [29], and phylogenetic relationships were reconstructed using FastTree2 (version 2.1.10) [30]. Taxonomic assignment was performed using the classify-sklearn naïve Bayes classifier against the SILVA Release 138 database. ASVs assigned to chloroplasts, mitochondria, eukaryotes, or other non-target lineages were removed before downstream analysis. The resulting filtered ASV table was used for subsequent diversity, taxonomic composition, Venn, heatmap, persistence, and differential-abundance analyses.

### 2.5. Diversity, Taxonomic Composition, and Group Comparisons

Rarefaction curves based on Chao richness, Shannon diversity, and Good’s coverage were generated to evaluate sequencing completeness. These curves were used to assess sampling sufficiency rather than to test formal group-level alpha-diversity differences. Beta diversity was assessed using Bray–Curtis dissimilarity calculated from ASV relative abundance profiles. Principal coordinate analysis (PCoA) was used to visualize compositional relationships among the seven sample groups, and overall group differences were evaluated using analysis of similarities (ANOSIM) with 999 permutations. Statistical significance was defined as *p* < 0.05.

To evaluate specific growth-stage differences, pairwise PERMANOVA based on Bray–Curtis dissimilarity was performed separately among the three gill-associated groups and among the three skin-mucus groups. Exact permutation probabilities were used because of the small sample size, and Benjamini–Hochberg correction was applied across the planned pairwise comparisons. Because gill-associated and skin-mucus samples with the same replicate number originated from the same pooled subset of three fish, Bray–Curtis dissimilarity was additionally calculated for each matched gill–skin pair. These paired dissimilarities were compared among growth stages using the Kruskal–Wallis test, followed by pairwise exact Mann–Whitney tests with Benjamini–Hochberg correction.

Taxonomic composition was summarized at the phylum, class, order, family, and genus levels based on relative abundance. Group-level differences in relative abundance at the phylum, genus, and ASV levels were evaluated using the Kruskal–Wallis H test. To control for multiple comparisons, Benjamini–Hochberg false-discovery-rate correction was applied separately to the complete set of tests at each taxonomic level. Both nominal *p* values and FDR-adjusted q values were reported. Taxa with q < 0.05 were considered statistically supported, whereas taxa with nominal *p* < 0.05 but q ≥ 0.05 were interpreted cautiously as exploratory candidates.

### 2.6. Classification of Transient, Intermittent, and Persistent ASVs

To characterize occurrence patterns across the complete dataset, the prevalence of each ASV was calculated across all 34 samples as the proportion of samples in which that ASV was detected. ASVs were classified as transient when prevalence was <0.2, intermittent when prevalence was ≥0.2 and ≤0.8, and persistent when prevalence was >0.8. Under this definition, persistent ASVs were detected in at least 28 of the 34 samples. These thresholds were defined a priori as operational prevalence categories to distinguish sporadically detected (<20%), intermediately prevalent (20–80%), and widely recurrent (>80%) ASVs across the complete dataset. With 34 samples, the >0.8 criterion required detection in at least 28 samples and therefore represented a stringent definition of dataset-wide recurrence. The term “persistent” is used here as an operational cross-sample prevalence category and does not imply longitudinal persistence within the same individual fish. After this dataset-wide classification, the ecological composition of each of the seven sample groups was summarized separately. For each group, the number of detected ASVs belonging to each prevalence category was expressed as a percentage of the total number of ASVs detected in that group, and the cumulative relative abundance contributed by ASVs in each category was calculated.

## 3. Results

### 3.1. Sequencing Sufficiency and Overall Community Differentiation

Rarefaction curves based on Chao richness increased rapidly with sequencing depth and subsequently approached plateaus, while Shannon diversity curves also stabilized and Good’s coverage values approached 1 across nearly all samples (Figure S1). These patterns indicated that sequencing depth was sufficient to capture the major detectable bacterial diversity in the analyzed communities. Considerable among-sample variation was visible in Chao and Shannon profiles, but no formal group-level alpha-diversity comparisons were inferred from the rarefaction curves.

**Figure 1:**
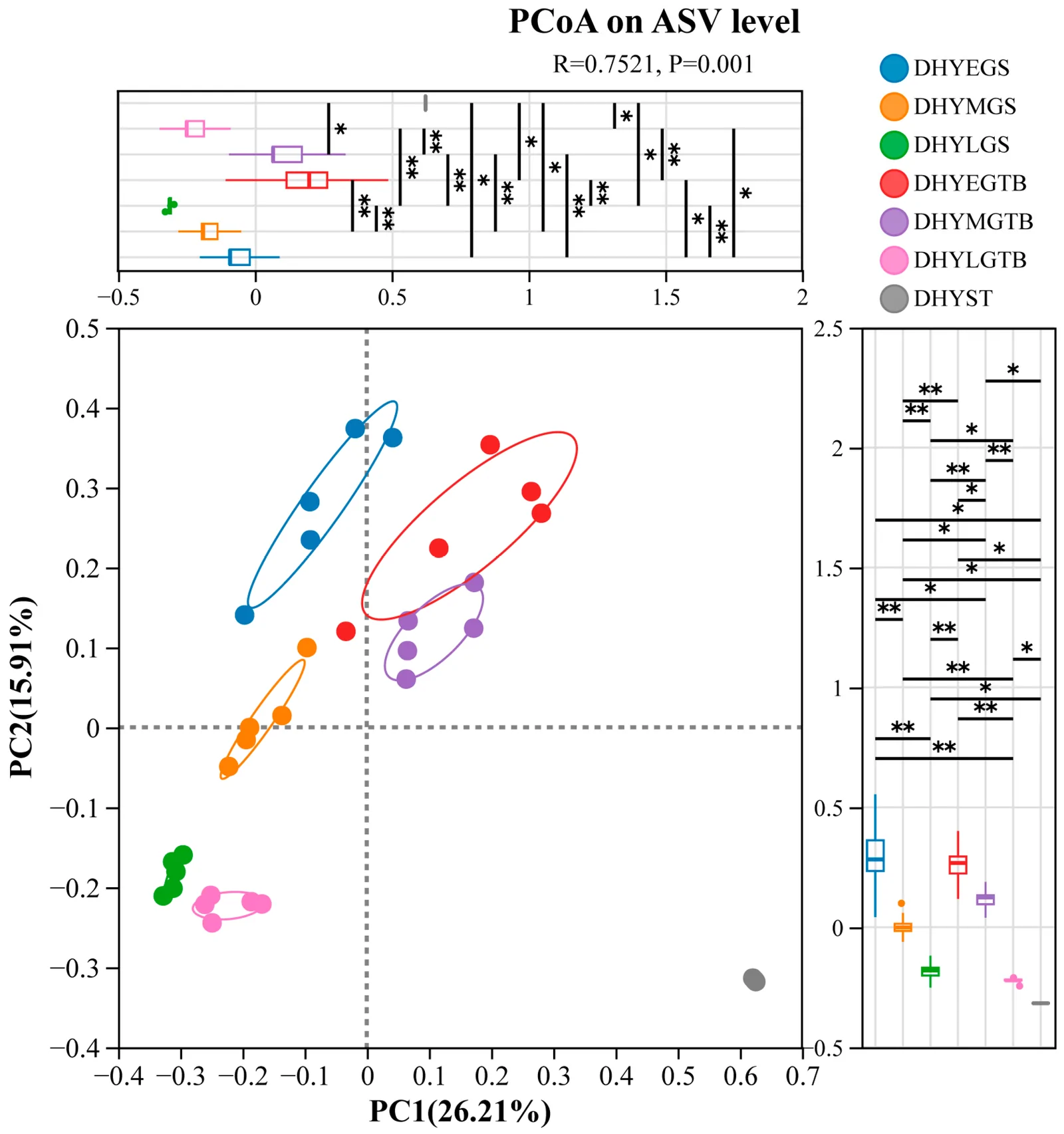
Principal coordinate analysis (PCoA) of microbial community structure across gill, skin-mucus, and supplied-seawater samples of *L. crocea*. Bray–Curtis dissimilarity was calculated from ASV relative abundance profiles. All four DHYST samples were included in the ordination; their symbols overlap closely because of highly similar coordinates in the first two PCoA dimensions. DHYEGS, DHYMGS, and DHYLGS represent gill samples from fish weighing 5–8 g, 39–46 g, and 160–162 g, respectively; DHYEGTB, DHYMGTB, and DHYLGTB represent the corresponding skin-mucus samples; DHYST represents supplied seawater. * *p* < 0.05, ** *p* < 0.01.

ASV-level PCoA revealed pronounced compositional differentiation among the seven sample groups (Figure 1). PC1 accounted for 26.21% of total community variation and PC2 accounted for 15.91%, together explaining 42.12%. ANOSIM confirmed significant overall differentiation among groups (R = 0.7521, *p* = 0.001). DHYEGS, DHYMGS, and DHYLGS occupied distinct regions of ordination space, consistent with growth-associated restructuring of the gill-associated microbiota. Skin mucus communities also differed across growth stages: DHYEGTB occupied a region clearly separated from DHYEGS, DHYMGTB shifted toward the central region, and DHYLGTB was positioned close to DHYLGS. Thus, the visual separation between gill and skin mucus was greatest at the early stage and reduced at the large stage. DHYST occupied a distinct region from all host-associated groups and showed comparatively tight clustering.

### 3.2. Taxonomic Reorganization Differed Between Gill, Skin Mucus, and Supplied Seawater

**Figure 2:**
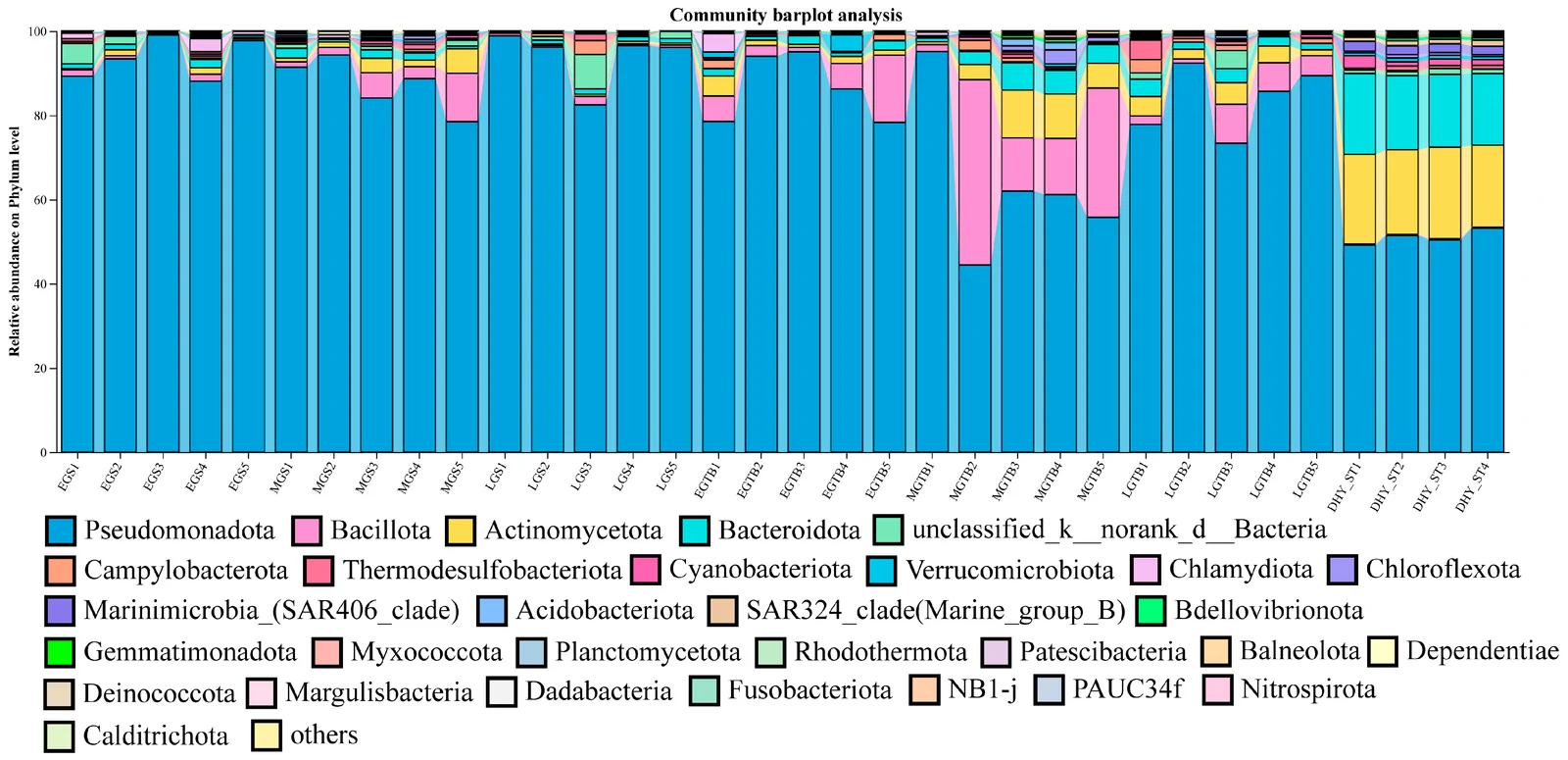
Phylum-level microbial community composition across gill, skin-mucus, and supplied-seawater samples of *L. crocea*. Stacked bars show the relative abundance of the dominant bacterial phyla in each sample.

*Pseudomonadota* represented the dominant phylum across most gill and skin mucus samples, although the relative contribution of secondary phyla varied markedly among growth stages and mucosal habitats (Figure 2). Early-stage gill samples were strongly dominated by *Pseudomonadota*, while middle-stage gill communities contained increased contributions from several secondary phyla. *Pseudomonadota* again accounted for a large proportion of DHYLGS communities. Skin mucus showed greater compositional heterogeneity across growth stages. *Pseudomonadota* remained dominant in DHYEGTB, but DHYMGTB contained larger and more variable contributions from *Bacillota*, *Actinomycetota*, *Bacteroidota*, and other bacterial lineages. By the largest stage, DHYLGTB again showed strong *Pseudomonadota* dominance, producing a broad phylum-level profile more similar to that of DHYLGS. The supplied seawater microbiota differed markedly from the host-associated communities. Although *Pseudomonadota* remained an important component, *Actinomycetota* and *Bacteroidota* made substantially larger contributions to DHYST than to most gill and skin mucus samples. Thus, the broad taxonomic framework of host-associated communities remained dominated by *Pseudomonadota*, whereas the environmental reference contained a more balanced distribution of several major phyla.

Class-level patterns further demonstrated variation among mucosal habitats and growth stages (Figure S2). *Gammaproteobacteria* and *Alphaproteobacteria* constituted major fractions of most host-associated communities. Early-stage skin mucus contained particularly high *Gammaproteobacteria* abundance, whereas *Alphaproteobacteria* became increasingly prominent in large-stage skin mucus. Several DHYMGTB samples contained increased contributions from Clostridia and Bacilli, consistent with the comparatively heterogeneous composition observed at the phylum level. In contrast, *Bacteroidia* and *Acidimicrobiia* contributed more strongly to the supplied seawater microbiota. At the order level, *Hyphomicrobiales*, *Burkholderiales*, *Enterobacterales*, *Pseudomonadales*, and *Rhodobacterales* represented major components of the host-associated microbiota (Figure S3). The relative contribution of these orders varied substantially among stages. *Hyphomicrobiales* and *Burkholderiales* were important in early-stage gills, whereas *Enterobacterales* and *Pseudomonadales* contributed strongly to several early and middle skin mucus communities. *Hyphomicrobiales* became increasingly prominent in both DHYLGS and DHYLGTB.

Family-level profiles revealed an even clearer distinction among sample groups (Figure S4). *Beijerinckiaceae*, *Vibrionaceae*, *Paracoccaceae*, *Moraxellaceae*, *Pseudomonadaceae*, *Comamonadaceae*, *Flavobacteriaceae*, *Burkholderiaceae*, *Lachnospiraceae*, *Rhizobiaceae*, and *Actinomarinaceae* represented important bacterial families. DHYEGS contained substantial *Beijerinckiaceae* and *Burkholderiales*-associated fractions, whereas DHYEGTB was characterized by high *Vibrionaceae* abundance. DHYMGS and especially DHYMGTB displayed greater among-sample heterogeneity, with different pools containing contrasting proportions of *Beijerinckiaceae*, *Vibrionaceae*, *Lachnospiraceae*, *Bacillaceae*, *Comamonadaceae*, and other families. In both DHYLGS and DHYLGTB, *Beijerinckiaceae* became increasingly prominent, further contributing to the reduced compositional separation between the two mucosal habitats at the largest stage.

### 3.3. Genus-Level Composition Revealed Distinct Gill and Skin Mucus Successional Trajectories

**Figure 3:**
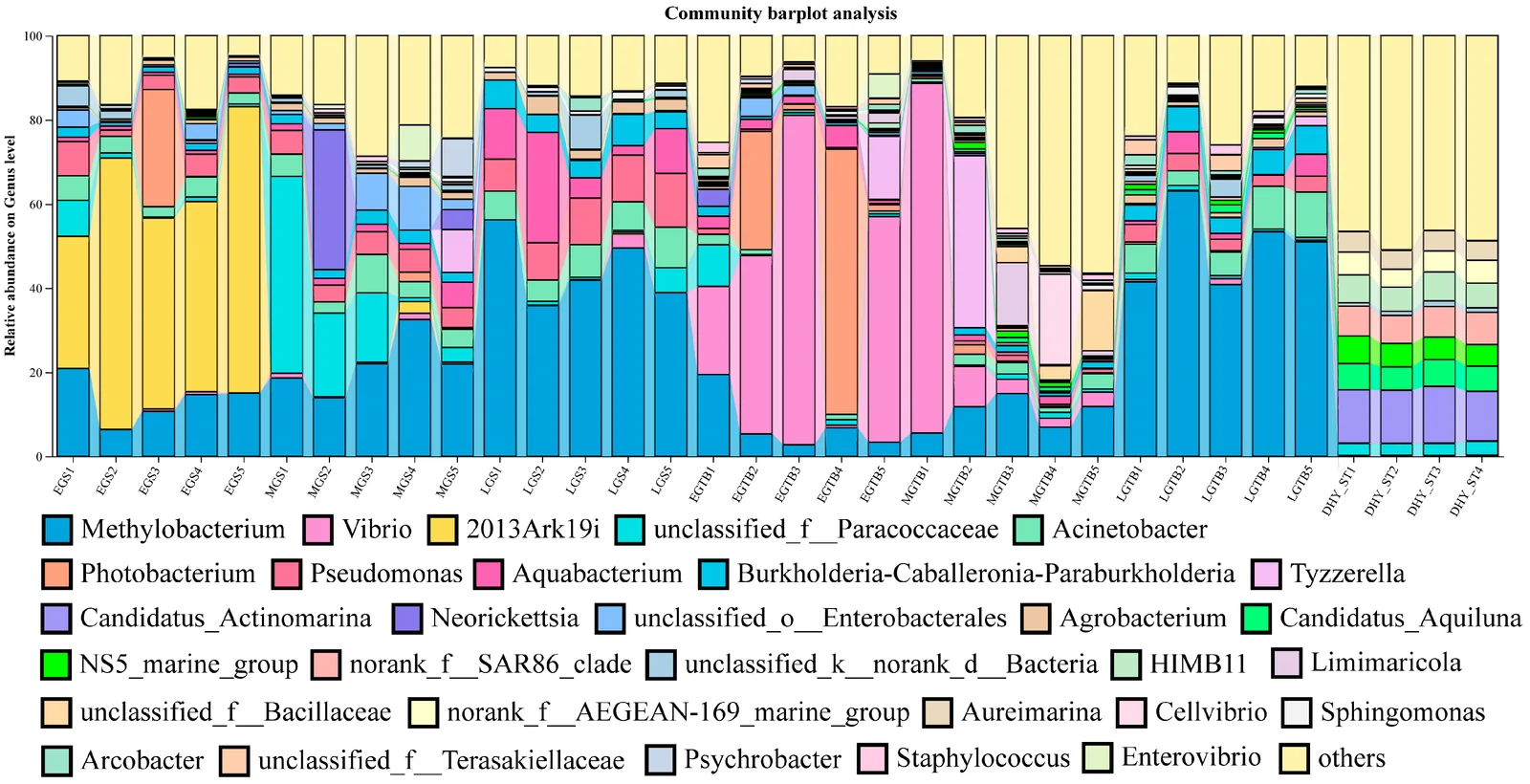
Genus-level microbial community composition across gill, skin-mucus, and supplied-seawater samples of *L. crocea*. Stacked bars show the relative abundance of the dominant genera or genus-level lineages in each sample.

Genus-level profiles showed substantially stronger differentiation among groups than was apparent at the phylum level (Figure 3). Dominant or recurrent genera included *Methylobacterium*, *Vibrio*, 2013Ark19i, unclassified *Paracoccaceae*, *Acinetobacter*, *Photobacterium*, *Pseudomonas*, *Aquabacterium*, *Burkholderia-Caballeronia-Paraburkholderia*, *Tyzzerella*, *Candidatus Actinomarina*, *Neorickettsia*, *Agrobacterium*, and *Candidatus Aquiluna*.

The early-stage gill-associated microbiota showed a characteristic taxonomic structure dominated by 2013Ark19i in several samples. This lineage declined strongly in the middle and large gill stages. DHYMGS displayed a more heterogeneous genus-level composition in which *Methylobacterium*, unclassified *Paracoccaceae*, *Acinetobacter*, *Pseudomonas*, *Aquabacterium*, and several additional genera contributed to different extents among pooled samples. A pronounced compositional change occurred in DHYLGS. *Methylobacterium* became one of the most abundant genera across the large-stage gill samples, whereas 2013Ark19i and several early-stage taxa contributed much smaller fractions. The large-stage gill-associated microbiota was therefore characterized by a redistribution of dominance rather than by simple retention of the early gill configuration.

Skin mucus displayed a different early-stage assemblage. DHYEGTB contained high relative abundances of *Vibrio*, and *Photobacterium* also contributed strongly to several samples. These taxa declined during subsequent growth, particularly in DHYLGTB. DHYMGTB exhibited the greatest replicate-to-replicate heterogeneity, with different samples containing varying combinations of *Vibrio*, *Tyzzerella*, *Methylobacterium*, and numerous lower-abundance genera. By the largest body-size stage, *Methylobacterium* increased markedly in DHYLGTB and became a major community component across all five pooled samples. The simultaneous increase in *Methylobacterium* in DHYLGS and DHYLGTB provides a taxonomic basis for the reduced separation of the two large-stage mucosal groups observed in the PCoA.

DHYST retained a distinct environmental taxonomic profile. *Candidatus Actinomarina*, *Candidatus Aquiluna*, NS5 marine group, SAR86-related lineages, HIMB11, AEGEAN-169 marine group, and other marine-water-associated taxa contributed much more strongly to the supplied seawater than to the host-associated samples. These patterns further emphasized that the external mucosal communities did not simply reproduce the composition of the supplied seawater microbiota.

Although the supplied seawater and host-associated communities shared several broad bacterial lineages, their finer-scale taxonomic profiles remained clearly differentiated. *Candidatus Actinomarina*, *Candidatus Aquiluna*, NS5 marine group, SAR86-related lineages, HIMB11, and AEGEAN-169 marine group contributed more strongly to DHYST than to most host-associated samples. These observations indicate that continuous exposure to an environmental microbial source did not result in direct compositional equivalence between the supplied seawater and external mucosal microbiota. However, because DHYST represented supplied source seawater rather than contemporaneous stage-matched tank water, these comparisons provide environmental context but cannot quantify stage-specific host–water microbial exchange.

### 3.4. Broad Taxonomic Overlap Contrasted with Extensive ASV-Level Turnover

Because corresponding gill-associated and skin-mucus samples originated from the same pooled fish subsets, paired Bray–Curtis dissimilarities were used to quantify compositional separation between the two mucosal habitats at each growth stage. Mean paired gill–skin dissimilarity was 0.869 ± 0.100 at the early stage, 0.824 ± 0.057 at the middle stage, and 0.407 ± 0.060 at the large stage (Kruskal–Wallis *p* = 0.0060). Dissimilarity at the large stage was significantly lower than that at both the early and middle stages after FDR correction (q = 0.0119 for both comparisons), whereas the early and middle stages did not differ significantly (q = 0.222). Thus, the reduced gill–skin separation observed in the ordination was quantitatively supported.

**Figure 4:**
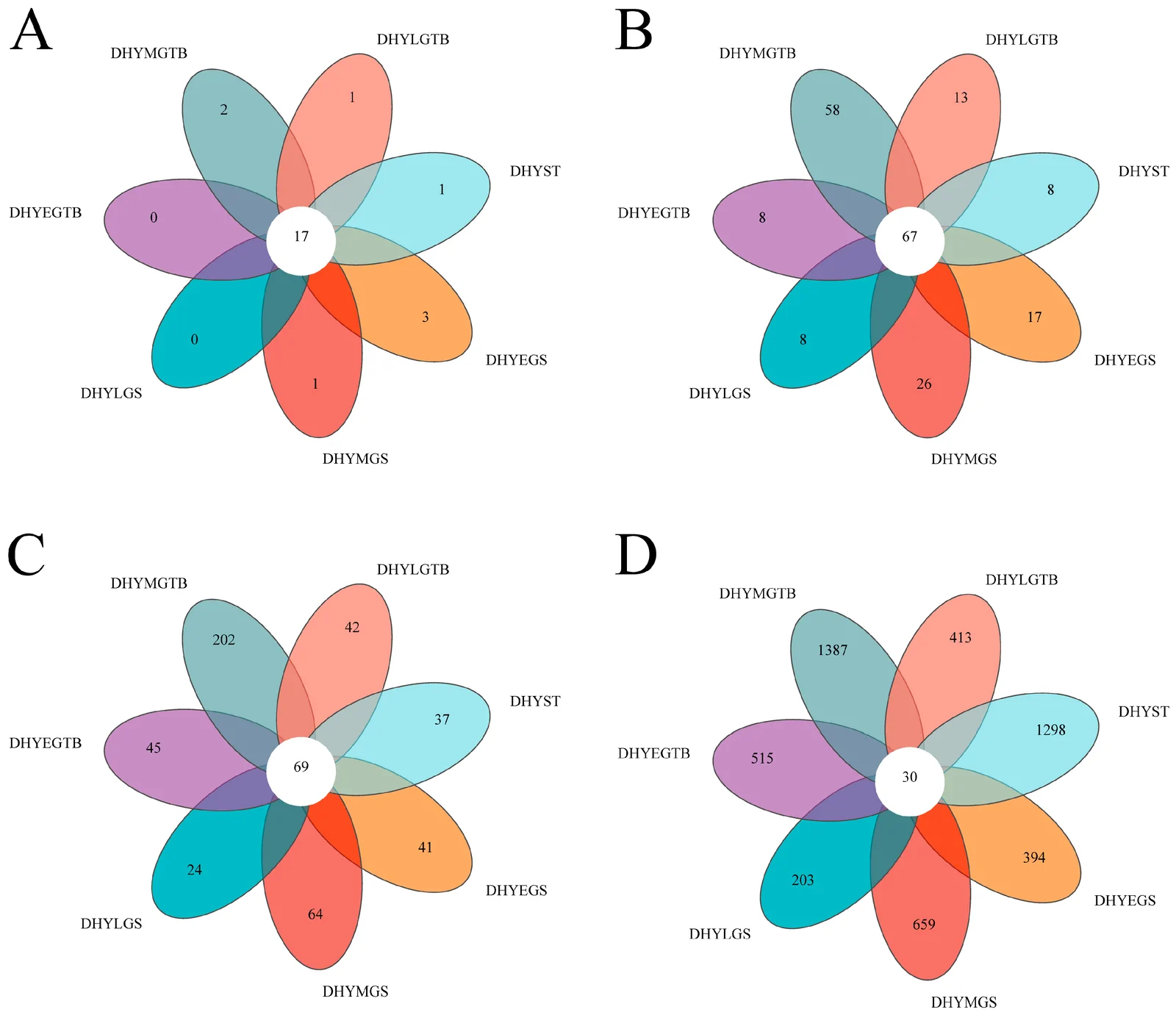
Shared and group-specific microbial taxa across gill, skin-mucus, and supplied-seawater samples of *L. crocea*. Venn diagrams show the numbers of shared and group-specific taxa at the (**A**) phylum, (**B**) family, (**C**) genus, and (**D**) ASV levels. A taxon was considered present in a group when it was detected in at least one sample assigned to that group.

Venn analysis demonstrated that the apparent degree of community overlap declined strongly with increasing taxonomic resolution (Figure 4). Seventeen phyla were shared among all seven groups, indicating extensive conservation at the broadest taxonomic level. Similarly, 67 families and 69 genera were detected across all groups. In contrast, only 30 ASVs were shared among all seven groups. The numbers of group-specific ASVs were 394 in DHYEGS, 659 in DHYMGS, 203 in DHYLGS, 515 in DHYEGTB, 1387 in DHYMGTB, 413 in DHYLGTB, and 1298 in DHYST. Thus, substantial overlap at the phylum, family, and genus levels coexisted with pronounced differentiation at the ASV level.

Among the host-associated groups, DHYMGTB contained the largest number of group-restricted ASVs, with 1387 ASVs detected exclusively within this group under the permissive criterion of occurrence in at least one pooled sample. Examination of within-group prevalence showed that most of these ASVs were infrequent: 1203 (86.73%) occurred in only one of the five DHYMGTB pools, 122 (8.80%) occurred in two pools, 61 (4.40%) occurred in three pools, and one ASV (0.07%) occurred in four pools; none occurred in all five pools. Nevertheless, 62 group-restricted ASVs were detected in at least three of the five DHYMGTB pools. Thus, the large group-restricted ASV pool primarily reflects pronounced fine-scale turnover and among-pool heterogeneity rather than a uniformly shared middle-stage-specific microbiota.

### 3.5. Persistent ASVs Became Increasingly Dominant in Later-Stage Gill and Skin Mucus Communities

**Figure 5:**
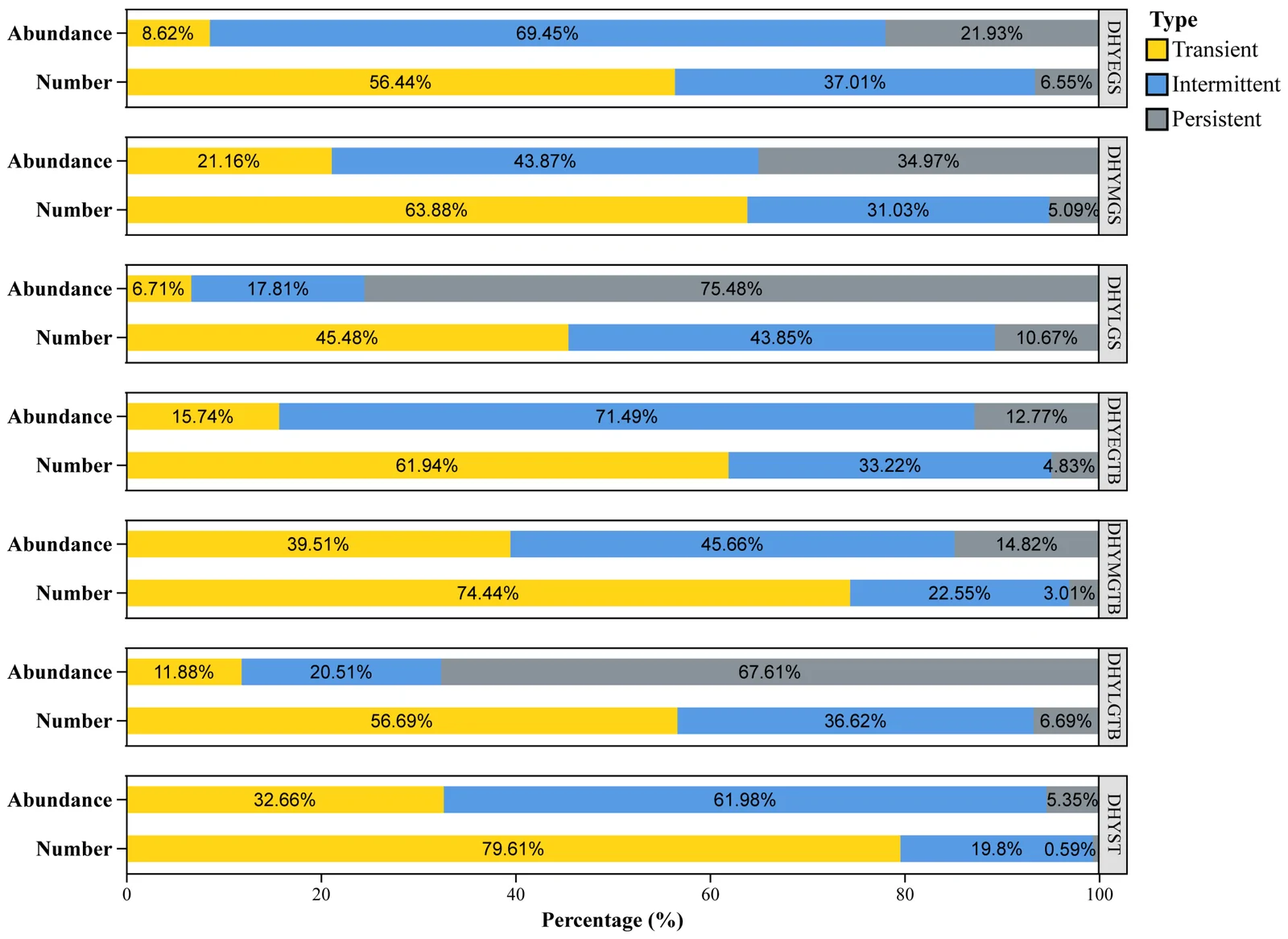
Relative representation and abundance contribution of transient, intermittent, and persistent ASVs across gill, skin-mucus, and supplied-seawater groups of *L. crocea*. ASV prevalence was calculated across all 34 samples. ASVs were classified as transient (prevalence < 0.2), intermittent (0.2 ≤ prevalence ≤ 0.8), or persistent (prevalence > 0.8). For each group, “Number” indicates the percentage of detected ASVs assigned to each prevalence category, whereas “Abundance” indicates the cumulative relative abundance contributed by ASVs in each category.

Dataset-wide occurrence classification revealed pronounced differences between the numerical representation and abundance contribution of transient, intermittent, and persistent ASVs (Figure 5). In DHYEGS, transient ASVs represented 56.44% of the detected ASVs, intermittent ASVs represented 37.01%, and persistent ASVs accounted for only 6.55%. Despite this small numerical fraction, persistent ASVs contributed 21.93% of total community abundance, while intermittent ASVs contributed 69.45% and transient ASVs contributed 8.62%. The relative abundance contribution of persistent ASVs increased during gill succession. In DHYMGS, persistent ASVs represented only 5.09% of the detected ASVs but contributed 34.97% of total abundance. Intermittent ASVs accounted for 31.03% of ASV number and 43.87% of abundance, whereas transient ASVs represented 63.88% of detected ASVs and contributed 21.16% of abundance. The clearest shift occurred in DHYLGS. Persistent ASVs represented only 10.67% of ASV number but contributed 75.48% of total community abundance. In contrast, transient ASVs still represented 45.48% of the detected ASVs but contributed only 6.71% of abundance. Intermittent ASVs represented 43.85% of ASV number and 17.81% of abundance. Thus, gill succession was accompanied by a strong increase in the quantitative contribution of persistent ASVs, despite their continued numerical minority.

Skin mucus displayed a more discontinuous pattern. In DHYEGTB, transient ASVs represented 61.94% of ASV number and 15.74% of total abundance, whereas persistent ASVs represented only 4.83% of ASV number and 12.77% of abundance. Intermittent ASVs contributed 71.49% of community abundance. DHYMGTB differed strongly from both the early and large skin mucus groups. Transient ASVs represented 74.44% of the detected ASVs and contributed 39.51% of total abundance, the highest transient abundance contribution among the six host-associated groups. Persistent ASVs represented only 3.01% of ASV number and 14.82% of abundance. Together with the exceptionally high number of group-specific ASVs, this pattern identified DHYMGTB as a community characterized by extensive fine-scale turnover. A pronounced shift occurred in DHYLGTB. Persistent ASVs represented only 6.69% of ASV number but contributed 67.61% of total abundance. Transient ASVs remained numerically dominant at 56.69% but contributed only 11.88% of abundance, while intermittent ASVs represented 36.62% of ASV number and 20.51% of abundance.

The supplied seawater showed a contrasting occupancy pattern. Transient ASVs represented 79.61% of detected ASVs, whereas persistent ASVs represented only 0.59%. Persistent ASVs contributed only 5.35% of total abundance, while intermittent ASVs contributed 61.98%. Because DHYST represents supplied seawater rather than stage-matched water communities, these values provide an environmental reference rather than a developmental trajectory.

Taken together, the persistence analysis revealed two contrasting but related mucosal patterns. Gill communities showed a relatively gradual increase in persistent-ASV abundance from early to large body-size stages, whereas skin mucus passed through a pronounced transient-rich intermediate state before persistent ASVs became quantitatively dominant in the largest fish.

### 3.6. Dominant ASVs Showed Habitat- and Growth-Stage-Associated Distributions

**Figure 6:**
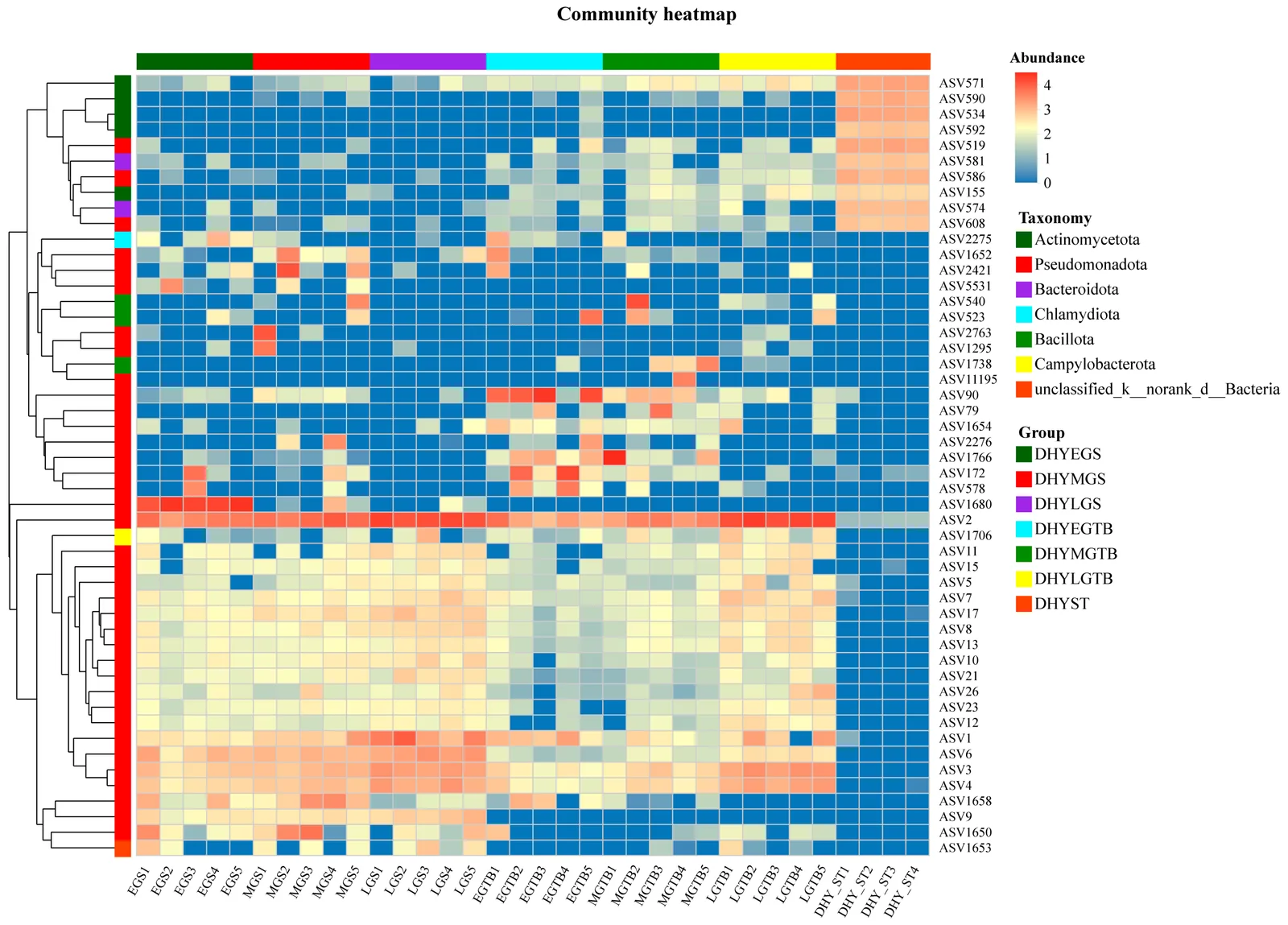
Heatmap of the 50 most abundant ASVs across gill-associated, skin-mucus, and supplied-seawater samples of *L. crocea*. Columns represent individual samples and rows represent ASVs. Color intensity represents log10-transformed ASV abundance. ASVs were hierarchically clustered using the average-linkage method, whereas samples were retained in their predefined order without sample-level clustering. The side annotation indicates ASV taxonomy at the phylum level, and the top annotation indicates sample group.

The Top-50 ASV heatmap further illustrated fine-scale differentiation among sample groups (Figure 6). A group of ASVs including ASV571, ASV590, ASV534, ASV592, ASV519, ASV581, and several related variants showed comparatively high abundance in DHYST but low representation in most host-associated samples.

Conversely, several host-associated ASVs displayed clear group-level patterns. ASV1680 was strongly represented in DHYEGS, ASV90 in DHYEGTB, and ASV1766 in DHYMGTB. Several ASVs, including ASV2, ASV1, ASV3, ASV4, and ASV6, contributed more strongly to later-stage host-associated communities. These distributions were consistent with extensive fine-scale turnover during growth and with the taxonomic replacement observed at the genus level. The heatmap also emphasized the distinction between the supplied seawater and host-associated communities. Multiple water-associated ASVs formed a cluster with consistently higher abundance in DHYST, whereas the dominant host-associated ASVs showed markedly different distributions across gill and skin mucus samples. Thus, the most abundant ASVs were not uniformly distributed across habitats but instead displayed strong sample-group associations.

### 3.7. Dominant Genera and ASVs Showed Distinct Group-Associated Abundance Patterns

**Figure 7:**
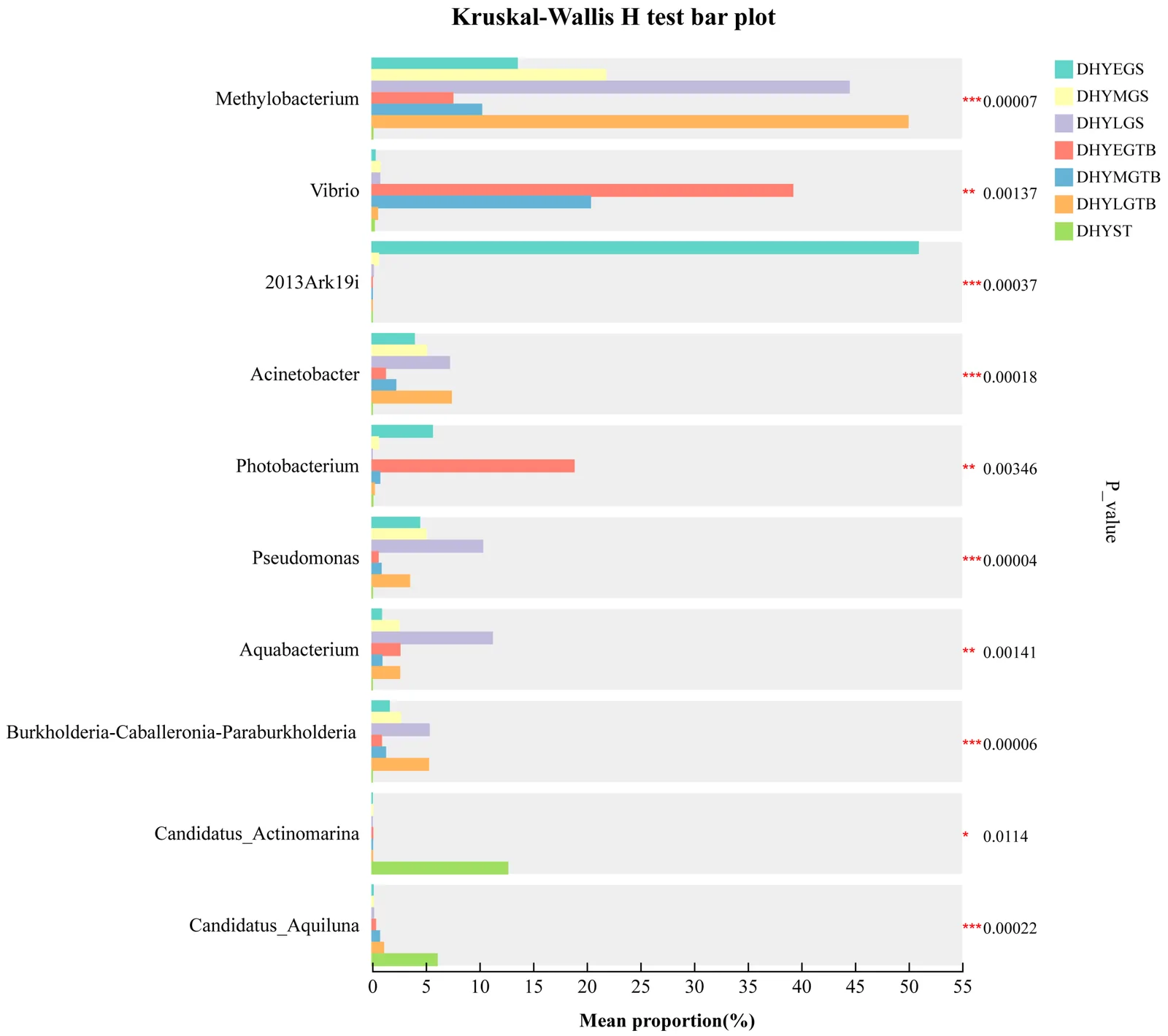
Group-associated abundance patterns of selected bacterial genera across gill-associated, skin-mucus, and supplied-seawater groups of *L. crocea*. Bars show mean relative abundance, with error bars representing standard deviation among pooled samples. Group differences were evaluated using the Kruskal–Wallis H test followed by Benjamini–Hochberg FDR correction. Nominal *p* values and adjusted q values are reported in Supplementary Table S2. * *p* < 0.05, ** *p* < 0.01, *** *p* < 0.001.

Kruskal–Wallis analysis revealed group-associated differences at multiple taxonomic levels. Among 51 phyla tested, 18 remained significant after Benjamini–Hochberg FDR correction. Major phyla showing FDR-supported differences included *Pseudomonadota* (q = 0.0157), *Bacillota* (q = 0.0098), *Actinomycetota* (q = 0.0098), and *Bacteroidota* (q = 0.0134). At the genus level, 173 of 1091 tested genus-level taxa remained significant after FDR correction. Kruskal–Wallis analysis identified multiple genera showing nominal group-level differences among the seven sample groups (Figure 7). *Methylobacterium* showed one of the strongest overall group effects (*p* = 0.00007). Its relative abundance was comparatively low or moderate in several early-stage groups but increased substantially in DHYLGS and especially DHYLGTB. In contrast, 2013Ark19i was strongly concentrated in DHYEGS and showed a pronounced overall group effect (*p* = 0.00037). Its abundance decreased markedly in subsequent gill stages and remained low in the skin mucus and supplied seawater groups. *Vibrio* also varied strongly among groups (*p* = 0.00137), reaching its greatest mean relative abundance in DHYEGTB and remaining an important component of DHYMGTB before declining in DHYLGTB. *Photobacterium* displayed a broadly similar pattern and showed a nominal group-level difference (*p* = 0.00346). Additional genera showing nominal differences included *Pseudomonas* (*p* = 0.00004), *Aquabacterium* (*p* = 0.00141), *Acinetobacter* (*p* = 0.00018), and *Burkholderia-Caballeronia-Paraburkholderia* (*p* = 0.00006). These genera contributed differently among gill and skin mucus groups and further demonstrated that growth-associated succession involved redistribution among multiple bacterial lineages rather than replacement by a single dominant taxon.

The supplied seawater-associated genera *Candidatus Actinomarina* (*p* = 0.0114) and *Candidatus Aquiluna* (*p* = 0.00022) showed their greatest contributions in DHYST and remained comparatively uncommon in most host-associated samples, reinforcing the compositional distinction between the supplied seawater and mucosal microbiota.

ASV-level comparisons revealed similar fine-scale group-associated patterns (Figure S5). ASV1680 showed its highest abundance in DHYEGS (*p* = 0.00037), whereas ASV90 was concentrated primarily in DHYEGTB (*p* = 0.00139). ASV1766 reached its highest abundance in DHYMGTB (*p* = 0.00015), and ASV540 also showed increased abundance in this group (*p* = 0.02077). Several ASVs contributed more strongly to later-stage host-associated communities. ASV2 showed a pronounced group-level difference (*p* = 0.00006) and was highly represented in large-stage samples. ASV1 (*p* = 0.001), ASV3 (*p* = 0.00005), ASV4 (*p* = 0.00007), and ASV6 (*p* = 0.00003) also showed distinct distributions among groups. Together, the genus- and ASV-level results provided complementary evidence that external mucosal succession involved both broad redistribution of dominant genera and fine-scale replacement of individual microbial populations.

Pairwise PERMANOVA further supported growth-stage-associated community differentiation within both external mucosal habitats. All three pairwise comparisons among gill-associated growth stages and all three comparisons among skin-mucus growth stages remained significant after FDR correction (all q = 0.0079).

## 4. Discussion

### 4.1. Growth Stage Was Associated with Pronounced but Tissue-Specific Reorganization of External Mucosal Microbiota

The present study revealed substantial growth-associated restructuring of the gill and skin mucus microbiota of cultured *L. crocea*. This conclusion was supported by several independent features of the dataset, including strong overall separation in ASV-level PCoA, pronounced changes in dominant bacterial lineages, extensive ASV-level turnover, and marked differences in the abundance contribution of transient, intermittent, and persistent microbial populations. Developmental restructuring of fish-associated microbial communities has been reported previously in several teleost species, although most studies have focused on intestinal communities [14,15,16,17,18]. The present results extend this developmental perspective to two continuously exposed external mucosal habitats. Both the gill and skin mucus communities changed substantially across the sampled body-size sequence, indicating that external mucosal microbiota also remain dynamic during post-juvenile growth.

Several processes covary with fish growth and may contribute to these microbial changes. Increasing body size is accompanied by changes in feeding intensity, metabolic demand, epithelial physiology, immune function, mucus production, surface area, and cumulative environmental exposure [1,4,9,22]. These factors may collectively alter the local conditions experienced by microorganisms on host surfaces. However, body weight, cultivation duration, developmental physiology, and feeding quantity were not experimentally separated in the present design. The observed patterns therefore represent growth-stage-associated microbiome succession rather than a direct causal effect of body mass. In addition, detailed contemporaneous records of stocking density and water physicochemical conditions were unavailable for all three sampling periods, preventing quantitative separation of these environmental factors from growth-stage-associated variation.

Importantly, the gill and skin mucus did not follow identical trajectories. This distinction is consistent with their different biological functions and microhabitat characteristics. The gill is continuously perfused by water while performing gas exchange, ion regulation, acid-base homeostasis, osmoregulation, and nitrogenous waste excretion [5,12]. Skin mucus forms a continuously renewed external barrier and presents a different physicochemical and host-derived interface to microbial colonists [9,10]. Consequently, growth-related changes in the host are expected to interact differently with these two mucosal habitats rather than producing a uniform response across the external surface.

The aquatic environment represents an important microbial source pool for external fish surfaces because both the gill and skin are continuously exposed to surrounding water. Nevertheless, host-associated communities need not passively reproduce environmental microbiota because local epithelial conditions, mucus characteristics, nutrient availability, and host-derived filtering can modify microbial recruitment and persistence [31]. In the present study, supplied seawater remained clearly differentiated from both gill-associated and skin-mucus communities, particularly at finer taxonomic resolutions. This pattern is consistent with selective host-surface assembly rather than simple passive reflection of the environmental microbial pool. However, because DHYST consisted of four independent supplied-seawater deliveries rather than stage-matched tank-water samples, the present dataset cannot quantify temporal microbial exchange between the culture water and host surfaces.

### 4.2. Strong Early Gill-Skin Differentiation Was Followed by Reduced Separation at the Largest Body-Size Stage

One of the clearest features of the dataset was the strong difference between gill and skin mucus microbiota in the smallest fish. DHYEGS and DHYEGTB occupied widely separated regions of the PCoA and were characterized by contrasting dominant taxa. Early gill communities contained high contributions from 2013Ark19i and ASV1680, whereas early skin mucus was characterized by high abundances of *Vibrio*, *Photobacterium*, ASV90, and other populations. This differentiation is consistent with the existence of strong tissue-associated ecological structuring. Previous studies across marine and cultured fishes have similarly demonstrated that gill, skin, gut, and environmental microbial communities can remain compositionally distinct even when they experience the same surrounding water [5,6,9,10,11,13,18]. Such patterns likely reflect differences in epithelial structure, mucus chemistry, oxygen exposure, nutrient availability, local host secretions, and immune conditions.

The relationship between the two external mucosal habitats changed with growth. DHYMGS and DHYMGTB remained clearly differentiated, but both groups differed substantially from their early-stage counterparts. By the largest stage, DHYLGS and DHYLGTB occupied much more closely positioned regions of the ordination space. This visual reduction in separation was also accompanied by increasingly similar taxonomic profiles, particularly the strong representation of *Beijerinckiaceae* and *Methylobacterium* in both large-stage groups.

Several non-exclusive processes may contribute to this pattern. Repeated environmental exposure may favor bacterial populations capable of occupying multiple mucosal surfaces. Alternatively, changes in mucus composition, epithelial physiology, host immune regulation, or nutrient availability during growth may alter the ecological contrast between gill and skin habitats. These mechanisms remain unresolved because the present study did not measure mucosal chemistry, immune status, epithelial properties, or colonization dynamics directly.

The important point is therefore not that the two habitats became identical, but that their relationship changed during growth. Strong early differentiation was followed by a later-stage community configuration in which the two external mucosal habitats shared more similar dominant bacterial structures.

### 4.3. Middle-Stage Skin Mucus Represented the Most Dynamic Host-Associated Community

The middle-stage skin mucus microbiota was distinguished by several independent features that collectively indicate unusually extensive microbial turnover. DHYMGTB contained 1387 group-specific ASVs, substantially more than any other host-associated group. At the same time, transient ASVs represented 74.44% of the ASV pool and contributed 39.51% of total community abundance. This combination is particularly informative because a high number of transient ASVs by itself could simply reflect many low-frequency detections. In DHYMGTB, however, transient populations were not only numerically abundant but also contributed a substantial fraction of total community abundance. Thus, the intermediate skin mucus community contained a large set of bacterial populations that were restricted in dataset-wide occurrence yet quantitatively important within this group. The exceptionally large group-restricted ASV pool in DHYMGTB should be interpreted in light of within-group prevalence. Most of these ASVs were detected in only one pooled sample, indicating that the high group-specific richness primarily reflects substantial fine-scale heterogeneity and turnover rather than a large set of uniformly shared stage-specific organisms. Nevertheless, 62 group-restricted ASVs recurred in at least three of the five DHYMGTB pools, indicating that a smaller but reproducible component was also associated with this intermediate-stage skin-mucus configuration.

The taxonomic composition data support the same interpretation. DHYMGTB displayed substantial replicate-to-replicate heterogeneity at multiple taxonomic levels, and the genus-level profiles contained varying combinations of *Vibrio*, *Tyzzerella*, *Methylobacterium*, and numerous less abundant lineages. ASV1766 and ASV540 also showed their greatest abundance in DHYMGTB. Together, these patterns indicate that the intermediate body-size class was not simply positioned between early and large stages but represented a distinct microbial configuration. Developmental transitions in fish-associated microbiota can include periods of pronounced restructuring as host development, environmental exposure, and local microbial recruitment change simultaneously [14,15,16]. Temporal and developmental changes in mucosal microbiota have also been reported in marine fishes, indicating that host-associated surface communities can remain highly dynamic rather than progressing monotonically toward a single stable configuration [6,9,18].

In the present study, DHYMGTB appears to represent such a high-turnover state. Numerous bacterial populations were detected specifically or preferentially at this stage, whereas relatively few belonged to the persistent category. This pattern is consistent with the high ecological plasticity reported for fish skin-associated microbial communities, which are continuously exposed to the surrounding aquatic environment while also being influenced by host-specific mucosal conditions [9,10,18]. The biological basis for this pattern remains unresolved. Changes in mucus production and renewal, epithelial properties, immune regulation, feeding intensity, body-surface characteristics, and cumulative exposure to environmental microorganisms may all contribute to variation in external mucosal microbiota during host growth [1,6,9,18]. Because these variables were not measured directly in the present study, they represent plausible ecological explanations rather than demonstrated causal mechanisms.

Taken together, DHYMGTB represented a transient-rich and compositionally heterogeneous intermediate configuration. The unusually large pool of group-restricted ASVs, the high transient-ASV abundance contribution, and marked among-pool taxonomic variability jointly support extensive fine-scale turnover at this sampling stage. Because mucus chemistry, immune variables, epithelial physiology, and contemporaneous environmental conditions were not measured, the mechanisms underlying this pattern remain unresolved.

### 4.4. Gill and Skin Mucus Differed in the Timing of Persistent-Community Dominance

Although persistent ASVs became increasingly important in both mucosal habitats, the timing of this change differed between gill and skin mucus. In the gill, the abundance contribution of persistent ASVs increased progressively from 21.93% in DHYEGS to 34.97% in DHYMGS and 75.48% in DHYLGS. This pattern indicates a relatively gradual redistribution of community abundance toward ASVs repeatedly detected across the complete dataset. Similar developmental restructuring of fish-associated microbiota has been reported across multiple teleost species, although the direction and magnitude of such changes vary according to host species, tissue type, and environmental context [6,14,16]. Skin mucus showed a different trajectory. Persistent ASVs contributed only 12.77% of abundance in DHYEGTB and remained low at 14.82% in DHYMGTB before increasing sharply to 67.61% in DHYLGTB. Thus, persistent-population dominance emerged later and more abruptly in skin mucus than in the gill.

This contrast reinforces the conclusion that the two external mucosal habitats undergo distinct growth-associated trajectories. The gill represents a physiologically specialized and continuously water-exposed mucosal surface involved in gas exchange, osmoregulation, ion transport, acid–base regulation, and nitrogenous waste excretion [5,12]. Skin mucus, in contrast, forms a dynamically renewed external barrier whose microbial community is influenced by both host-associated conditions and continuous environmental exposure [9,10,18]. These contrasting tissue properties provide a plausible ecological context for the more gradual persistence pattern observed in the gill and the pronounced intermediate-stage turnover observed in skin mucus.

The most important feature of the persistence results is the distinction between numerical representation and quantitative dominance. Even in DHYLGS and DHYLGTB, persistent ASVs represented only 10.67% and 6.69% of detected ASVs, respectively. Nevertheless, they contributed 75.48% and 67.61% of total community abundance. Similar discrepancies between microbial occurrence and quantitative dominance are consistent with the broader concept that host-associated microbial communities contain both recurrent populations and a large, more variable low-frequency fraction [1,2,7,8]. Later-stage community organization therefore did not involve elimination of transient populations. Large numbers of transient and intermittent ASVs remained detectable, but community abundance became increasingly concentrated within a comparatively small persistent fraction. Such redistribution is compatible with host-associated microbiome assembly in which repeated environmental exposure, local habitat conditions, and host-related filtering favor a subset of recurrent microbial populations [2,7].

### 4.5. Broad Taxonomic Conservation Concealed Extensive Fine-Scale Population Replacement

A major feature of the dataset was the strong dependence of community overlap on taxonomic resolution. Seventeen phyla, 67 families, and 69 genera were shared among all seven groups, whereas only 30 ASVs were universally shared. This contrast demonstrates that relatively conserved higher-level taxonomic composition can coexist with extensive fine-scale microbial turnover. Developmental succession therefore did not require complete replacement of dominant phyla or genera. Instead, much of the differentiation occurred among individual ASVs nested within broadly shared higher taxonomic groups.

Similar patterns have been increasingly recognized in fish microbiome research. Microbial communities that appear broadly comparable at the phylum or genus level may contain substantially different fine-scale populations, reflecting variation associated with host species, mucosal habitat, developmental condition, and environmental exposure [6,7,8,10]. Our previous study of *L. crocea* likewise showed that differences among host tissues and rearing environments became increasingly evident at finer taxonomic resolution [25]. Such taxonomic-resolution dependence is ecologically important because microorganisms assigned to the same broad taxonomic lineage can differ substantially in environmental tolerance, colonization ability, substrate use, and host association [1,2,10]. Consequently, similarity at the genus level does not necessarily imply equivalent community organization or ecological behavior. *Methylobacterium* provides a clear example in the present study. The genus was detected before the largest stage but increased markedly in both DHYLGS and DHYLGTB. Thus, developmental restructuring involved not only gain and loss of bacterial populations but also substantial redistribution of abundance among taxa retained across multiple groups. Likewise, the Top-50 ASV heatmap showed pronounced group specificity even where higher-level taxonomic profiles were broadly similar. ASV1680, ASV90, ASV1766, and several later-stage ASVs displayed highly distinct distributions across groups. These fine-scale patterns help explain why substantial beta-diversity differentiation remained evident despite broad *Pseudomonadota* dominance across many host-associated samples.

Fine-scale variation within broadly conserved fish-associated microbial communities has also been documented across large comparative datasets, in which host biology, habitat, and environmental conditions explain substantial differences below coarse taxonomic levels [6,18]. The present results therefore reinforce the importance of interpreting developmental microbiome succession at multiple taxonomic resolutions. Higher-level composition defines the broad microbial framework, whereas ASV-level data reveal the population turnover and abundance redistribution underlying growth-associated restructuring.

### 4.6. Taxonomic and ASV-Level Patterns Reflect Growth-Associated Microbial Community Restructuring

Several bacterial genera showed clear group-associated abundance patterns and therefore provide useful taxonomic indicators of the observed succession. 2013Ark19i was strongly associated with DHYEGS and declined sharply at later stages, making it one of the clearest markers of the early gill-associated microbiota. In contrast, *Vibrio* and *Photobacterium* were particularly abundant in DHYEGTB and declined substantially in later-stage skin mucus. *Methylobacterium* followed the opposite pattern. It became increasingly prominent in large-stage gill and skin mucus communities and contributed strongly to the increasingly similar taxonomic structure of DHYLGS and DHYLGTB. *Aquabacterium*, *Acinetobacter*, *Pseudomonas*, and *Burkholderia-Caballeronia-Paraburkholderia* also showed substantial group-associated variation.

These patterns are consistent with previous studies demonstrating pronounced tissue-, habitat-, and environment-associated differentiation of fish mucosal microbiota [5,13,18]. In *L. crocea* specifically, distinct bacterial communities have previously been reported among the skin, gill, gut, and surrounding environment [13,25]. The taxa identified here nevertheless represent ecological indicators of community restructuring rather than demonstrated functional determinants. Amplicon sequencing based on the 16S rRNA gene primarily provides information on taxonomic composition and relative abundance and does not directly resolve microbial activity, strain-level functional variation, or causal effects on host physiology [2,4].

This limitation is particularly important when interpreting genera such as *Vibrio* and *Photobacterium*. Members of these genera occur widely in marine environments and fish-associated microbiomes, and their detection at genus level does not by itself establish pathogenicity or disease status [2,9]. Their relatively high abundance in DHYEGTB is therefore interpreted here as a taxonomic feature of the early skin-mucus community rather than evidence of disease. Similarly, the later-stage increase in *Methylobacterium* does not establish a beneficial or host-promoting role. Its importance in the current dataset lies in its strong association with the large-stage external mucosal microbiota and its contribution to the reduced taxonomic separation between DHYLGS and DHYLGTB. ASV-level results provide more specific ecological indicators. ASV1680 was associated with early gills, ASV90 with early skin mucus, ASV1766 with intermediate skin mucus, and ASV2 together with ASV1, ASV3, ASV4, and ASV6 contributed more strongly to later-stage host communities.

The greater specificity of ASV-level distributions is consistent with the observation that fine-scale microbial differentiation can remain substantial even when broader taxonomic groups are shared among host tissues and environments [8,10,25]. These ASVs therefore represent candidate markers of developmental microbiome restructuring, although their detailed taxonomy and biological functions cannot be resolved from the present amplicon dataset.

### 4.7. An Integrated View of Growth-Associated External Mucosal Microbiome Reorganization

Taken together, the results support a non-linear pattern of external mucosal microbiome reorganization in cultured *L. crocea*. At the smallest body-size stage, gill and skin mucus communities were strongly differentiated. This differentiation was visible in ordination space and was supported by contrasting dominant taxa, including 2013Ark19i in the gill and *Vibrio* and *Photobacterium* in skin mucus. Strong tissue-specific structuring of external mucosal microbiota has also been reported in other marine fishes, supporting the concept that gill and skin represent distinct microbial habitats despite continuous exposure to the same aquatic environment [5,11,13].

The intermediate stage represented a period of substantial restructuring, but this process was particularly pronounced in skin mucus. DHYMGTB contained the largest number of host-associated group-specific ASVs, the highest proportion of transient ASVs, and the greatest transient abundance contribution among host groups. Developmental and temporal restructuring of fish-associated microbiota has been documented in several teleost species, and these trajectories are frequently non-linear rather than simple transitions toward a universally stable adult community [14,15,17,18]. At the largest stage, the two mucosal habitats remained distinguishable but showed reduced separation in ordination space and increasingly similar dominant taxonomic structures. In both tissues, community abundance also became concentrated within a relatively small persistent ASV fraction. Persistent ASVs contributed 75.48% of abundance in DHYLGS and 67.61% in DHYLGTB.

The overall pattern can therefore be summarized as strong early mucosal differentiation, extensive intermediate-stage restructuring, and later-stage increasing dominance of persistent microbial populations accompanied by reduced gill–skin separation. This interpretation is consistent with the broader view that fish-associated microbiomes are shaped jointly by host development, tissue-specific habitat conditions, environmental exposure, and repeated microbial recruitment [2,6,9,18].

This interpretation remains bounded by the experimental design. The pooled samples capture variation among composite microbiome samples rather than among individual fish. Host physiological variables, mucus chemistry, immune parameters, epithelial properties, and environmental physicochemical conditions were not measured concurrently. Consequently, the present study identifies robust growth-associated ecological patterns but does not isolate the causal mechanisms responsible for them. The present data therefore indicate that growth-associated external mucosal microbiome succession in *L. crocea* involves not only replacement of bacterial populations but also substantial redistribution of ecological dominance between transient, intermittent, and persistent community members. Because different fish were sampled at each body-size stage, the present study represents repeated cross-sectional sampling of the same cultured population rather than longitudinal tracking of individual fish. Consequently, the observed patterns should be interpreted as growth-stage-associated community differences rather than within-individual microbial trajectories. Because the present study was based on 16S rRNA gene amplicon sequencing, microbial gene content, gene expression, and metabolic activity were not directly resolved. We therefore avoided assigning functional changes solely from taxonomic profiles. Future metagenomic, metatranscriptomic, and metabolomic analyses will be required to determine whether the growth-stage-associated taxonomic reorganization identified here is accompanied by corresponding changes in microbial functional potential or activity.

## 5. Conclusions

Gill-associated and skin-mucus microbiota of cultured *L. crocea* showed clear growth-stage-associated reorganization across the sampled body-size sequence. The two habitats were strongly differentiated at the early stage, middle-stage skin mucus exhibited pronounced fine-scale turnover, and later-stage communities showed increased abundance dominance of dataset-wide persistent ASVs. Paired community comparisons further demonstrated substantially reduced gill–skin compositional dissimilarity at the largest body-size stage. Supplied seawater remained compositionally distinct from host-associated communities, although the absence of stage-matched tank-water samples prevents inference of temporal host–water coupling. Overall, these results support tissue-specific and non-linear growth-stage-associated restructuring of external mucosal microbiota rather than a uniform developmental trajectory.

## Data Availability

The raw sequence data are available in the Genome Sequence Archive (Genomics, Proteomics & Bioinformatics 2021) at the National Genomics Data Center (Nucleic Acids Res. 2022), China National Center for Bioinformation/Beijing Institute of Genomics, Chinese Academy of Sciences (accession codes: CRA049855).

## Supplementary Materials

The following supporting information can be downloaded at https://www.mdpi.com/article/10.3390/biology15211922/s1: Figure S1: Rarefaction curves for (A) Chao richness, (B) Shannon diversity, and (C) Good’s coverage across gill, skin-mucus, and supplied-seawater samples of *L. crocea*; Figure S2: Class-level microbial community composition across gill, skin-mucus, and supplied-seawater samples of *L. crocea*. Stacked bars show relative abundance of the dominant bacterial classes; Figure S3: Order-level microbial community composition across gill, skin-mucus, and supplied-seawater samples of *L. crocea*. Stacked bars show relative abundance of the dominant bacterial orders; Figure S4: Family-level microbial community composition across gill, skin-mucus, and supplied-seawater samples of *L. crocea*. Stacked bars show relative abundance of the dominant bacterial families; Figure S5: Candidate ASVs showing nominal group-level differences across gill, skin-mucus, and supplied-seawater groups of *L. crocea*. Bars show mean relative abundance for each group. Overall group differences were evaluated using the Kruskal–Wallis H test. * *p* < 0.05, ** *p* < 0.01, and *** *p* < 0.001; Table S1: Complete Kruskal–Wallis H-test results and Benjamini–Hochberg FDR-adjusted q values for phylum-, genus-, and selected ASV-level comparisons across gill-associated, skin-mucus, and supplied-seawater groups of *L. crocea*; Table S2: Pairwise community-comparison statistics based on Bray–Curtis dissimilarity, paired gill–skin dissimilarity analyses across growth stages, and within-group prevalence distributions of group-restricted ASVs.
